# Artificial Intelligence in Uropathology

**DOI:** 10.3390/diagnostics14202279

**Published:** 2024-10-14

**Authors:** Katia Ramos Moreira Leite, Petronio Augusto de Souza Melo

**Affiliations:** Laboratory of Medical Investigation, Urology Department, University of São Paulo Medical School, LIM55, Av Dr. Arnando 455, Sao Paulo 01246-903, SP, Brazil; petroniomelo@usp.br

**Keywords:** uropathology, artificial intelligence, prostate cancer, bladder cancer, renal cell cancer, diagnosis, prognosis

## Abstract

The global population is currently at unprecedented levels, with an estimated 7.8 billion people inhabiting the planet. We are witnessing a rise in cancer cases, attributed to improved control of cardiovascular diseases and a growing elderly population. While this has resulted in an increased workload for pathologists, it also presents an opportunity for advancement. The accurate classification of tumors and identification of prognostic and predictive factors demand specialized expertise and attention. Fortunately, the rapid progression of artificial intelligence (AI) offers new prospects in medicine, particularly in diagnostics such as image and surgical pathology. This article explores the transformative impact of AI in the field of uropathology, with a particular focus on its application in diagnosing, grading, and prognosticating various urological cancers. AI, especially deep learning algorithms, has shown significant potential in improving the accuracy and efficiency of pathology workflows. This comprehensive review is dedicated to providing an insightful overview of the primary data concerning the utilization of AI in diagnosing, predicting prognosis, and determining drug responses for tumors of the urinary tract. By embracing these advancements, we can look forward to improved outcomes and better patient care.

## 1. Introduction

Artificial intelligence (AI) is a transformative technology that enables computers and machines to replicate human-like cognitive functions, including learning, problem-solving, and decision-making. It involves the development of algorithms and systems capable of performing tasks traditionally requiring human intelligence, such as understanding natural language, identifying patterns, and making predictions. AI is proving to be a game-changer across various sectors, particularly in healthcare, where it streamlines processes, boosts efficiency, and facilitates data-driven decision-making.

A significant subset of AI is machine learning, which focuses on developing algorithms and statistical models that allow computers to learn from data and make predictions or decisions autonomously, without explicit programming. This approach empowers machines to enhance their performance by learning from experience and recognizing data patterns, rather than relying on predefined rules. Machine learning algorithms have found applications in various domains, such as image and speech recognition, natural language processing, recommendation systems, and predictive analytics, where they automate processes and extract valuable insights from vast amounts of data [1].

## 2. Deep Learning

Within machine learning, deep learning is dedicated to using artificial neural networks with multiple layers (deep neural networks) to tackle complex problems. Deep learning algorithms are designed to learn, extract, and represent hierarchical features from data, enabling them to make more sophisticated and accurate predictions or decisions. Through a process known as backpropagation, these models fine-tune their parameters to minimize errors and optimize performance on specific tasks [2].

Deep learning has demonstrated exceptional capabilities in image analysis, particularly in the domain of uropathology. It has shown remarkable success in medical image analysis, achieving state-of-the-art results in challenging tasks that were previously difficult for traditional machine learning approaches. Studies have shown that deep learning models can accurately classify histopathological images, distinguishing between benign and malignant lesions with a high degree of precision [3]. This capability is especially relevant in prostate cancer (PCa), where accurate grading of histological images can significantly impact treatment decisions.

The integration of diverse types of data, including clinical and genetic data, as well as radiological and histopathological images, enhances the learning capabilities of deep learning algorithms [2].

In anatomic pathology, deep learning has significantly advanced the field by enhancing the diagnostic capabilities of pathologists and improving predictions of patient outcomes. Specific applications of deep learning in anatomic pathology include the following:

1. Image analysis: Deep learning algorithms are trained on large datasets of histopathology slides to automatically detect and classify patterns and abnormalities. This enhances the workflow of pathologists, improving both the speed and accuracy of diagnoses. For example, in the detection of PCa, AI models have achieved diagnostic accuracy comparable to that of expert pathologists [4].
2. Disease detection and classification: Deep learning models assist pathologists in detecting and classifying various diseases based on image data, providing more objective and consistent diagnostic results. In addition to PCa, AI has been applied in the detection of other urological malignancies, such as bladder cancer, where deep learning models have shown promise in non-invasive diagnosis through urine cytology analysis [5].
3. Prognostic predictions: Deep learning techniques can analyze multiple data sources to predict patient outcomes and disease progression, supporting pathologists and healthcare providers in making more personalized treatment decisions. This is particularly important in uropathology, where the prognosis of diseases like renal cell carcinoma can vary widely based on molecular and histopathological features [6].
4. Resource optimization: Automation tools powered by deep learning streamline the workflow of pathologists, reduce manual tasks, and prioritize complex cases, potentially improving efficiency, reducing turnaround times, and alleviating the workload on pathologists. AI-driven systems can also help to identify cases that require further review by pathologists, thereby optimizing the allocation of resources in pathology departments [7].

Overall, deep learning has the potential to revolutionize anatomic pathology by enabling faster, more accurate diagnoses, personalized treatment strategies, and improved patient outcomes through the integration of advanced computational techniques with traditional pathology practices. Additionally, the global shortage of pathologists underscores the urgent need for AI. The declining number of pathologists worldwide over the past 20 years, coupled with challenges related to aging populations and an increasing burden of cancer, highlights the pressing necessity for innovative solutions like AI in uropathology [8].

## 3. AI in Prostate Cancer Diagnosis and Grading

Prostate cancer (PCa) is the most frequently diagnosed cancer among men in over half of the countries worldwide, with an estimated 1.4 million new cases in 2020. The numbers are expected to double by 2040. In the USA alone, approximately 1 million men undergo prostate biopsies annually, with at least 12 biopsy cores taken from each patient [9]. These figures highlight the substantial workload on pathologists, as biopsy results are crucial for treatment decisions and prognostic determinations. Research has shown promising results for AI in the digital diagnosis and grading of PCa. Various studies have demonstrated high accuracy in diagnosing and grading PCa using digital pathology images, with some reporting an area under the curve (AUC) as high as 0.99, indicating the significant potential of AI in uropathology [3,10,11,12].

The Gleason/ISUP score is a critical factor in predicting PCa prognosis. It is determined based on the architecture of the glands and has five prognostic grades: Gleason 6 (3 + 3) (ISUP 1), Gleason 7 (3 + 4) (ISUP 2), Gleason 7 (4 + 3) (ISUP 3), Gleason 8 (4 + 4) (3 + 5) (5 + 3) (ISUP 4), and Gleason 9 and 10 (ISUP 5) [13]. Gleason 6/ISUP 1 adenocarcinoma is generally managed with active surveillance (AS) due to its low malignant potential [14]. However, determining Gleason grading remains subjective and dependent on the pathologist’s experience, leading to inter-pathologist and intra-pathologist variability [15,16,17]. Several studies have explored AI-based methods for PCa grading. For example, Lucas et al. [18] found an AUC of 0.92 for distinguishing benign from malignant tissue and 0.90 for differentiating Gleason grade ≥4 from ≤3. Källen et al. [19] achieved an accuracy of 81% to 89% in classifying benign tissue and Gleason patterns using automated methods. Arvaniti et al. [20] compared an AI model’s diagnosis with that of pathologists, achieving agreements of 0.75 and 0.71. Nir et al. [21] trained classifiers on tissue microarray cores, showing an overall grading agreement of 0.51 with pathologists. Nagpal et al. [22] found 72% agreement with genitourinary (GU) pathologists and 58% with general pathologists. Ström et al. [10] demonstrated an AUC of 0.997 for distinguishing benign from malignant tissue and a mean kappa of 0.62 for Gleason grading. Bulten et al. [11] achieved an AUC of 0.999 for differentiating benign from malignant tissue and an AUC of 0.978 for differentiating ISUP score 1 from 2 or more.

We have conducted a study where 1525 image patches were analyzed from 12 slides of radical prostatectomy specimens, achieving an accuracy of 91.2% in the validation set using deep learning methods for cancer diagnosis. The model was able to recognize benign tissue and Gleason patterns 3, 4, and 5 with high accuracy (96.7%, 92.5%, 96.5%, and 76.6%, respectively) [23]. Researchers have also utilized various Application Programming Interfaces (APIs) to build systems for prostate image analysis. For example, Gifani and Shalbaf [24] tested 15 pre-trained convolutional neural networks (CNNs) and found that NasNetLarge performed better, achieving an accuracy of 0.93 and an AUC of 0.98 in classifying prostate images.

Several commercial programs are available for PCa diagnosis and grading in core biopsies. For example, the IBEX program has demonstrated high accuracy in cancer detection, with an AUC of 0.99, as well as in categorizing different cancer grades [3]. Paige, an FDA-approved program, has been shown to reduce evaluation time and the need for a second opinion and immunohistochemistry [25,26]. IRA MATRIX developed a deep learning-based algorithm called AIRAProstate for tumor detection and Gleason grading. They evaluated the reclassification of patients with ISUP 1 PCa in an AS program. AIRAProstate upgraded 33% of patients who later left the AS and only 8% of those who were not reclassified during follow-up, with an odds ratio (OR) of 3.3 (*p* = 0.04) [27].

Recently, we published a study aiming to evaluate and compare the performance of top-ranked public and commercial algorithms using real-world data. Using a diverse dataset of whole-slide prostate biopsy images with a range of Gleason scores from various sources, predictions were obtained from five top-ranked public algorithms from the Prostate cANcer graDe Assessment (PANDA) challenge and two commercial Gleason grading algorithms. The comparison was made with the evaluation of 10 uropathologists (UP). The pairwise quadratic weighted kappa among pathologists ranged from 0.777 to 0.916. Both public and commercial algorithms showed high agreement with pathologists, with quadratic kappa ranging from 0.617 to 0.900, proving that commercial algorithms performed on par or outperformed top public algorithms [28].

In summary, AI algorithms have been showing promising results, indicating that AI can match or even exceed human performance in PCa diagnosis and grading. This advancement points towards a future where AI could significantly enhance the accuracy and efficiency of PCa management.

## 4. AI in Prostate Cancer Prognosis

Prognostication is crucial for making informed treatment decisions in non-metastatic PCa. Adjuvant androgen deprivation therapy (ADT) combined with radiotherapy can benefit patients with localized PCa. However, ADT can negatively impact the quality of life, leading to a need for careful patient selection. AI-based systems offer the potential to predict patient prognosis, therapeutic response, and risk of recurrence or progression, thereby improving treatment planning.

AI has shown significant promise in enhancing the accuracy and personalization of prognostic models for PCa. For instance, a U.S. population-based cohort study of men diagnosed with non-metastatic PCa demonstrated that the algorithm “Survival Quilts” had good discrimination for predicting 10-year prostate-cancer-specific mortality, performing similarly to other multivariable models like PREDICT Prostate and the MSKCC nomograms [29]. This highlights the growing potential of AI in prognostic modeling for PCa, offering an additional layer of precision in guiding treatment strategies. The integration of AI models with clinical data, such as PSA levels, Gleason scores, and imaging findings, could further refine prognostication, enabling clinicians to tailor treatment approaches more effectively.

Johns Hopkins University developed a deep learning method based on visual analysis using tissue microarray (TMA) spots from patients, 72% of whom represent recurrence cases. This deep learning system demonstrated a strong association with recurrence in the test set of the cohort, with an OR of 3.28 (95% CI 1.73–6.23; *p* < 0.005) per unit increase. The biomarker maintained a strong correlation even after adjusting for ISUP grade, PSA level at diagnosis, positive surgical margins, and prostatectomy year. In the multivariate analysis, the AI biomarker was strongly associated with recurrence, with a hazard ratio (HR) of 3.02 (CI 1.10–8.29; *p* = 0.03) per unit increase [30]. This finding suggests that AI can complement traditional histopathological assessments by providing quantitative insights that may improve the accuracy of recurrence predictions.

In another study, Spratt et al. used digital pathology images from pretreatment prostate tissue along with clinical data from 5727 patients treated with radiotherapy, with or without ADT, to identify those who would benefit most from ADT. The endpoint was the development of metastasis. Validation in another cohort of 1594 patients showed that 34% were identified by the model as positive for benefiting from ADT, and ADT significantly reduced the risk of distant metastasis compared with radiotherapy alone [31]. This study illustrates how AI can be leveraged to optimize therapeutic strategies, ensuring that patients receive the most effective treatment while minimizing unnecessary side effects.

Furthermore, AI algorithms have been developed to predict not only recurrence but also other critical outcomes such as metastasis. For example, a recent study explored the use of CNNs to predict lymph node metastasis (LNM) directly from primary tumor histology [32]. The study involved analyzing hematoxylin and eosin (H&E)-stained slides from 218 patients who underwent radical prostatectomy. The CNN, trained on these images, achieved a mean AUC of 0.68 and a balanced accuracy of 61.37%, indicating its potential utility in predicting LNM. Furthermore, the study demonstrated that the CNN probability score was an independent predictor of LNM, alongside lymphovascular invasion. These findings suggest that CNN-based image analysis could serve as a novel, low-cost method for extracting relevant prognostic information directly from histological images, contributing to more accurate lymph node status predictions in PCa patients.

While AI tools for routine PCa diagnostics and prognostication show considerable promise, their widespread implementation remains limited. Several factors contribute to this, including the high costs associated with digital pathology workflow, the absence of clear regulatory guidelines for AI deployment in clinical practice, and the lack of prospective studies demonstrating the real-world benefits of AI algorithms. Addressing these challenges will be essential for the successful integration of AI into clinical workflows, ultimately improving outcomes for patients with PCa.

In conclusion, AI shows promise in improving PCa treatment decisions by accurately predicting prognosis, recurrence, and therapy response, despite challenges in widespread implementation.

## 5. AI in Urothelial Carcinoma

Grading and staging, identifying histological subtypes, assessing lymphovascular invasion, and detecting carcinoma in situ are crucial for risk classification and management of urothelial cancer (UC) patients [33]. Recent advancements in AI have shown significant promise in improving the accuracy and consistency of these tasks.

For instance, Zhang et al. developed an algorithm to diagnose papillary UC, achieving a true-positive rate of 0.95 in discriminating between normal and tumor tissue [34]. This level of accuracy underscores the potential of AI to assist in the early detection of UC. Similarly, Noorbakhsh et al. achieved an impressive AUC of 0.98 in distinguishing normal tissue from tumors, further demonstrating the high diagnostic potential of AI in urothelial carcinoma [35].

In the context of grading, AI systems have shown varying degrees of success. Studies have reported accuracy ranges from 79% to 88% in grading UC. For example, Jansen et al. conducted a study involving 328 transurethral resections of bladder UC, comparing an AI model with the diagnoses of three UP. The AI algorithm correctly graded 76% of low-grade and 71% of high-grade cancers, achieving moderate consensus with pathologists (kappa = 0.48), which was comparable to the agreement among pathologists themselves (kappa = 0.35, 0.38, and 0.52) [36]. These findings suggest that while AI can match human performance in grading UC, there is still room for improvement.

Yin et al. developed an algorithm to stage bladder cancer by analyzing the shape, size, and color of the nucleus and cytoplasm. Their model distinguished between pTa and pT1 stages with an accuracy of 96%. However, the study has limitations, as it excluded challenging cases that represent real-world complexities often encountered by experimented pathologists [37].

Identifying muscle invasion in bladder cancer is critical for determining the need for radical treatment, such as radical cystectomy, often following neoadjuvant chemotherapy. Sarkar et al. developed a deep learning model to differentiate non-muscle-invasive (NMIBC) from muscle-invasive bladder cancer (MIBC). The model achieved an accuracy of 79.72%, with a sensitivity of 66.62%, a specificity of 87.39%, and a precision of 75.58% [38]. To further enhance diagnostic accuracy, Pan et al. demonstrated that an AI model could achieve an AUC of 0.878 (95% CI 0.875–0.881) at the patch level and 0.870 (95% CI 0.805–0.923) using the whole-slide image for assessing the depth of invasion and histologic grade in bladder cancer. These results were comparable to the average diagnostic level of pathologists, with an AUC of 0.847 (95% CI 0.779–0.905) [39].

Additionally, Garcia et al. tested an AI algorithm’s efficacy in detecting invasive growth patterns (such as nodular, trabecular, and infiltrative) of MIBC, which have been shown to predict patient outcomes. Their immunohistochemistry-based algorithm accurately discriminated between these growth patterns, achieving an average accuracy of 90% [40]. This capability could provide valuable prognostic information, potentially guiding treatment decisions.

While neoadjuvant treatment for high-risk bladder cancer remains a standard of care, selecting appropriate patients is crucial due to the potential delays in surgery and the high morbidity associated with the treatment, especially in frail patients. Although existing models use tomography images to predict treatment response, there is currently a gap in histopathology-based models for this purpose, highlighting an area where AI could make a significant impact in the future.

In summary, recent advancements in artificial intelligence (AI) have shown promising results in improving the diagnosis, grading, and staging of urothelial cancer (UC). Although AI’s performance in grading UC has varied, its utility in staging and identifying muscle invasion has been notable, with models distinguishing between stages and invasion types with high accuracy and sensitivity. Furthermore, AI’s potential in predicting treatment responses in high-risk bladder cancer highlights its significant impact on the management and treatment decision-making for UC patients.

## 6. AI in Renal Cell Cancer

The 5th edition of the WHO/IARC blue books introduces over 30 types of renal tumors, with additional types awaiting more detailed descriptions, referred to as provisional [41]. Tumor classification in renal cell carcinoma (RCC) is based on histopathological characteristics, often supported by specific genetic alterations, which are crucial for prognosis and therapy planning. The classification of RCC is particularly important because the behavior of these tumors ranges from indolent to highly aggressive. Despite the importance of accurate classification, studies utilizing AI for RCC classification remain limited.

One example of AI application in this field is the study of papillary clear cell renal cell tumor (pccRCT), an indolent variant of RCC that closely resembles clear cell renal cell carcinoma (ccRCC). Differentiating between these two can be challenging for pathologists, typically requiring immunohistochemical analysis. However, a recent study demonstrated that AI could distinguish ccRCC from pccRCT with accuracies of 90% and 91%, respectively, suggesting that AI could become a valuable tool in assisting pathologists with difficult differential diagnoses [42].

There are two primary grading systems for RCC: the WHO/ISUP system, which is based on nucleoli prominence, and the Fuhrman grade, which considers the size and shape of the nucleus [43]. The distinction between low-grade (Fuhrman grades 1 and 2) and high-grade (Fuhrman grades 3 and 4) RCC is critical for determining prognosis and treatment strategies. Yeh et al. developed an automated system that distinguishes between low-grade and high-grade RCC, reporting a false-positive rate of 0.2, a true-positive rate of 1.0, and an AUC of 0.97 using their model [44]. This high level of accuracy highlights the potential of AI in enhancing the consistency and reliability of RCC grading.

Additionally, Holdbrook et al. conducted a similar evaluation, incorporating nucleoli prominence into their analysis. Their study demonstrated a good correlation (0.59) between the AI system and a multigene score assay-based system, which is a recognized prognosticator in RCC [45]. This suggests that AI models can not only replicate but also potentially enhance current grading practices by integrating additional histopathological features.

Furthermore, Tian et al. utilized data from The Cancer Genome Atlas (TCGA) to develop a method that predicted RCC grade with 84.6% sensitivity and 81.3% specificity in the test set. Importantly, they were able to associate the AI-predicted grade with overall survival, even after adjusting for variables such as age and gender, with a hazard ratio of 2.05 (95% CI 1.21–3.47) [46]. This finding underscores the potential of AI not only in grading but also in providing prognostic information that could guide clinical decision-making.

In summary, the significant expansion of renal tumor types emphasizes the role of histopathological and genetic characteristics in the prognosis and treatment of RCC. Studies on AI applied to RCC classification show promising results, also proving valuable in grading, and even predicting overall survival rates. These advancements highlight AI’s potential to support pathologists in diagnosis, grading, and treatment planning for RCC.

## 7. AI as a Predictive Biomarker

Decades of research have led to significant advancements in the treatment of cancer patients, including the development of immunotherapies and targeted therapies. Immune checkpoint inhibitors, for example, have significantly improved survival rates in patients with advanced RCC [47]. Tumor mutation burden (TMB) has emerged as an important biomarker for predicting the response to immune checkpoint blockade across various cancers [48]. In a study by Marostica et al. [49], the authors demonstrated that histopathology images could be used to predict TMB in ccRCC, with a Spearman correlation coefficient of 0.419 between true and predicted TMB values (Spearman correlation test, *p* = 0.0003). This finding suggests that AI has the potential to identify molecular features associated with treatment response directly from histological images.

The expression of PD-L1 serves as a critical biomarker for predicting responses to anti-PD1/PD-L1 inhibitors and is utilized as a companion diagnostic test essential for the prescription of these drugs. However, manual quantification of PD-L1 expression can be time-consuming and is often characterized by significant inter- and intra-observer variability. In their analysis of multiple tumor types, including urothelial carcinoma, Baxi et al. [43] demonstrated that AI used for the evaluation of PD-L1 expression outperformed manual scoring methods. Their study, which included data from the CheckMate clinical trials, showed that AI algorithms identified more PD-L1-positive cases compared to manual scoring, particularly at cutoffs of ≥1% and ≥5%. Additionally, the AI-powered scoring demonstrated improved associations with patient survival in certain tumor types, highlighting its potential to enhance the accuracy and consistency of biomarker assessment in clinical practice [50] in detecting positive PD-L1 cases, which consistently correlated with both response and survival.

Tyrosine-kinase inhibitors (TKIs) have also improved outcomes for patients with metastatic RCC (mRCC), but predictive markers for TKI responsiveness are still lacking. Go et al. [51] developed a machine learning-based classifier to predict responsiveness to VEGFR-TKI in mRCC patients. This classifier, which was based on clinical, laboratory, histopathological, immunohistochemical, mutational, and miRNA features, achieved an accuracy of 0.87, indicating a promising approach to personalizing treatment for mRCC patients. The integration of AI in this context could lead to more precise and tailored therapeutic strategies, ultimately improving patient outcomes.

In conclusion, groundbreaking advancements in RCC treatment, such as immunotherapies and targeted therapies, have significantly improved survival rates. AI has demonstrated potential in predicting TMB and assessing PD-L1 expression, offering more accurate identification of patients who would benefit from treatment and improving survival. Furthermore, machine learning has been utilized to predict responsiveness to VEGFR-TKI in mRCC patients with high accuracy, indicating a move towards personalized treatment strategies that could enhance patient outcomes.

## 8. Discussion

Implementing AI in surgical pathology is a complex and multifaceted endeavor. While AI applications in pathology have made remarkable progress, there remain significant challenges in establishing and maintaining such facilities. Key obstacles include ensuring access to cutting-edge technology, which remains unevenly distributed globally, and addressing the substantial infrastructure and expertise required to deploy AI effectively. It is important to note that when implementing AI, certain factors need to be considered. These include the need for high-quality annotated datasets for training AI models, ensuring variability in histopathological slides due to differences in staining and scanning techniques, and subjecting the models to rigorous validation, including internal, external, and real-time validation, which are often lacking in current studies. The opaque nature of AI models is also a challenge that affects the credibility of current models. To address these challenges, a multidisciplinary approach is required to ensure that they are safe and effective [52,53].

The regulatory landscape for AI is complex and evolving. Regulations must balance innovation with safety to ensure that AI tools are effective and reliable. Current frameworks are being updated to address AI’s unique characteristics, such as algorithm transparency and data quality control. Regulatory compliance is crucial, and clear guidelines are needed to facilitate safe AI adoption. Economically, AI has the potential to improve productivity and reduce costs, but if poorly implemented, it may increase workload and exacerbate disparities. The economic value of AI must be assessed through comprehensive frameworks that consider its impact on health outcomes and costs. Successful AI integration requires training healthcare professionals to understand and effectively use AI tools. This includes enhancing AI literacy among pathologists to ensure they can interpret AI outputs and integrate them into clinical decision-making [54,55].

Despite the challenges, the potential benefits of AI are substantial. AI has the capacity to significantly enhance the diagnostic accuracy, grading, and prognostic assessments made by pathologists. Moreover, it can aid in the identification of new predictive biomarkers, which could transform patient care. Given the potential rewards, the effort to overcome these challenges and fully integrate AI into pathology is not only justified but essential for the future of precision medicine.

## Data Availability

Not applicable.
